# Does colour-morph variation in metabolic physiology and oxidative stress match morph-specific life–history strategies?

**DOI:** 10.1007/s00442-025-05728-x

**Published:** 2025-05-26

**Authors:** Chiara Morosinotto, Antoine Stier, Suvi Ruuskanen, Natacha Garcin, Patrik Karell

**Affiliations:** 1 Department of Biology, Lund University, Ecology Building, SE-223 62 Lund, Sweden; 2 Novia University of Applied Sciences, Faculty of Bioeconomy, FI-10600 Ekenäs, Finland; 3 Department of Biology, University of Padova, 35121 Padova, Italy; 4 National Biodiversity Future Center (NBFC), 90133 Palermo, Italy; 5 Department of Biology, University of Turku, FI-20014 Turku, Finland; 6 Univ Lyon, Université Claude Bernard Lyon 1, CNRS, ENTPE, UMR 5023 LEHNA, F-69622 Villeurbanne, France; 7 Institut Pluridisciplinaire Hubert Curien, UMR7178, Université de Strasbourg, CNRS, Strasbourg, France; 8 Department of Biological and Environmental Science, University of Jyväskylä, Jyväskylä, Finland

**Keywords:** mtDNA copy number, Thyroid hormones, Melanin, Colour polymorphism, Life stages

## Abstract

Understanding to what extent phenotypes vary in their physiological traits and their associations to life-history strategies may help to better understand how animals are adapted to their environment and how they can cope with changing conditions. Melanin-based colour polymorphism is a phenotypic trait closely associated with physiological characteristics and fitness, which in tawny owls (*Strix aluco*) is highly heritable and strongly associated with adult survival. Pheomelanic (brown) tawny owl adults raise heavier offspring, suggesting higher parental effort and/or faster growth of brown offspring, but have shorter lifespan than grey ones. Moreover, brown morphs show faster rate of telomere shortening than the grey morph, but only after reaching adulthood. To further explore the potential physiological mechanisms being involved in such trade-offs, we aimed at characterizing markers of metabolic physiology (thyroid hormones and mitochondrial density) and oxidative stress (reactive-oxygen metabolites) between brown and grey tawny owls, both at the nestling and adult stages. Although there was no significant effect of colour morph on thyroid hormones or mitochondrial density, brown nestlings had higher oxidative damage levels than grey individuals. Conversely in adults, mitochondrial density was higher in brown individuals, without a significant impact on oxidative stress levels. Morph-specific differences in physiological traits are thus life-stage dependent, but seem to match morph-specific life-history strategies since the higher oxidative stress observed in brown nestlings could result from their faster growth, while the higher mitochondrial density of brown adults could help in supporting their higher reproductive effort.

**Supplementary Information:**

The online version contains supplementary material available at 10.1007/s00442-025-05728-x.

## Introduction

Understanding the physiological mechanisms underlying phenotypic variation and life-history strategies can help us understand how animals adapt to the current and future environmental conditions (Ricklefs and Wikelski 2002). The physiological mechanisms underlying life-history trade-offs, thereby shaping life-history strategies, may be widespread across a majority of taxa since trade-offs are fundamental in the ecology of any species (review in Monaghan et al. 2010; Hood et al. 2018; Husak and Lailvaux 2022). Among many proposed mechanisms, telomere dynamics, mitochondrial metabolism and oxidative stress have been suggested as widespread mechanisms underlying senescence patterns and life-history trade-offs at the cellular level (review in Costantini 2008; Monaghan et al. 2009; Monaghan et al. 2010; Hood et al. 2018; Koch et al. 2021; Tobler et al. 2021; Husak and Lailvaux 2022). Higher reproductive effort and faster growth are energetically demanding and have been suggested to request higher mitochondrial metabolism, efficiency and/or number to allow animals to produce the necessary ATP (Garrattet al. 2013; Salin et al. 2019; Quéméneur et al. 2022). Yet, higher mitochondrial metabolism can have the potential cost of higher reactive-oxygen species (hereafter ROS) production which, if not efficiently counteracted by antioxidant mechanisms, can lead to oxidative stress (Costantini 2008). The damage caused by oxidative stress within the cells plays a major role in aging processes and oxidative stress can lead to faster telomere shortening and can ultimately lower survival (Costantini et al. 2010; Monaghan 2014; Reichert and Stier 2017). Other important coordinators of energetics are thyroid hormones that are metabolic hormones associated with several physiological traits and processes (Kim 2008; Elliott et al. 2013), such as development, thermoregulation, and growth (Ruuskanen and Hsu 2018), but also contribute to coordination of timing of life-history events (e.g. Perez et al. 2016). In their role as coordinators of thermogenesis and metabolism, thyroid hormones (hereafter TH) stimulate mitochondrial biogenesis (Harper and Seifert 2008), usually inducing an increase in oxidative stress levels (Venditti and Di Meo 2006; Rey et al. 2014) and generally higher THs increase metabolism across taxa (e.g. Ruuskanen et al. 2021)

All these biomarkers could thus be linked with the reproduction–survival and growth–survival trade-offs (Passos et al. 2007; Monaghan et al. 2009; Monaghan et al. 2010; Smith et al. 2016; Monaghan and Ozanne 2018; Kochet al. 2021; Quéméneur et al. 2022). Species displaying melanin-based colour polymorphism are an ideal system to investigate the links between variation in physiological biomarkers, phenotype, and life–history traits, because colour morphs are expected to be differently adapted to environmental conditions (Roulin et al. 2004). Differential melanin production in the morphs is expected to covary with various physiological functions (Ducrest et al. 2008), which in turn are expected to lead to differential resource allocation and thereby life–history decisions (Roff 1996).

The tawny owl (*Strix aluco*) has two melanin-based colour morphs, a grey and a brown, depending on the level of pheomelanin in the feathers (Brommer et al. 2005). Colour morphs are highly heritable and not dependent on age, sex or environmental conditions (Brommer et al. 2005, Karell et al. 2011a, Morosinotto et al. 2020). Previous studies showed that the two morphs vary substantially in several life–history traits. Brown owls have heavier offspring that grow faster in good environmental conditions (Piault et al. 2009, Morosinotto et al. 2020), and provide more consistent parental care (Emaresi et al. 2014). At the northernmost geographical limit, grey owls live longer (Brommer et al. 2005) because of survival selection against the brown morph, which fluctuates depending on the harshness of winter conditions (Karell et al. 2011a). This differential survival could be linked to different thermoregulation abilities, since grey owls have more dense, insulating plumage (Koskenpato et al. 2016), better camouflage abilities in winter (Koskenpato et al. 2020), and the two morphs seem to maintain different body temperature under extreme winter conditions (Authors unpublished results). Furthermore, adult brown tawny owls have shorter telomeres and faster telomere shortening (Karell et al. 2017), although this difference is not present among offspring (Morosinotto et al. 2021), suggesting that in adults these telomere dynamics derive from accumulating maintenance costs during the breeding life span. Morphs also vary in their immune response, with adult brown owls mounting a stronger immune response but also paying higher costs in terms of body mass loss (Gasparini et al. 2009; Karell et al. 2011b).

Here we analysed how thyroid hormones (T3 and T4), mitochondrial density (i.e. mtDNA copy number, which correlates with aerobic metabolism) and oxidative state (i.e. reactive-oxygen metabolites) vary in wild tawny owl adults and offspring belonging to the two morphs. We expect the two morphs to have substantially different physiological profiles, with individuals of the brown morph having higher thyroid hormone levels, mitochondrial density and oxidative stress because of their faster pace of life characterized by their faster growth as nestlings (Piault et al. 2009; Morosinotto et al. 2020), higher reproductive effort as adults (Emaresi et al. 2014), as well as their generally shorter lifespan at northern latitudes where this study takes place (Brommer et al. 2005). We also expect sexes to differ in their physiological profile (Costantini 2018) due to the substantial size dimorphism in this species, with females being larger than males, and different reproductive effort during parental care in this species.

## Methods

Tawny owls were sampled between 2016 and 2019 from a well-established nest-box population in western Uusimaa, Southern Finland (60˚15’ N, 24˚ 15’ E) that has been monitored since 1979 (*ca.* 200 nest boxes in 500 km^2^; Karell et al. 2009, Karell et al. 2011a, Morosinotto et al. 2020). All nest boxes were checked for nests and clutch size, brood size and hatching date were recorded for any breeding attempt. Owing to nest failures, nest predation events and logistic field work constraints we were not able to catch and sample all adult breeding owls in the population (see ‘Statistical methods’ below). In successful nests adults were trapped, measured and ringed within few days after hatching (Karell et al. 2009) and plumage colour was scored as either grey or brown morph from the colouration estimated in facial disc, breast, back and overall colouration (see details in Brommer et al. 2005). At approximately 25 days old (age estimated from wing length; due to the strong hatching asynchrony in this species offspring within the same nest may have few days of age difference), all offspring were ringed, weighed and colour scored (Morosinotto et al. 2020). From each indvidual, both adults and offspring, two blood samples were collected at ringing from the brachial vein for laboratory analyses, one used for DNA extraction and one to separate plasma and red blood cells (see below).

### Laboratory analyses

#### Mitochondrial density

One blood sample per individual was stored in ethanol (2016–2018) or SET buffer (2019) and kept at − 20°C. DNA was extracted from all the samples using ammonium acetate (NH_4_Ac) in 2019 at Lund University, the protocol used was a modification from (Nicholls et al. 2000), see details in Morosinotto et al. (2021, 2022). All offspring samples were then sexed using a PCR-based method with a 99.1% success rate, following a modified protocol from Kekkonen et al. (2008), see details in Morosinotto et al. (2021) and Tooth et al. (2024). DNA purity for all samples was evaluated with Nanodrop (see details in Morosinotto et al. 2021) and all the DNA samples used in the mitochondrial density protocol were diluted in water to reach a concentration of 2ng μl^-1^.

Relative mitochondrial DNA copy number (mtDNAcn) was measured as an index of mitochondrial density with qPCR as previously used in various avian species (Stier et al. 2019; Velando et al. 2019; Cossin-Sevrin et al. 2022). mtDNAcn has been shown to be moderately to strongly correlated to mitochondrial respiration rate in both nestlings and adult passerine birds (Stier et al. 2019; Cossin-Sevrin et al. 2022). The single-copy and ultra-conserved sfsr/3 sequence was used as the nuclear single-copy reference gene (sfsr/3Fb 5’-ACTAGCCCTTTCAGCGTCATGT-3’ and sfsr/3Rb 5’-CATGCTCGGGAACCAAAGG-3’) as previously used in this species for telomere length normalization (Karell et al. 2017; Morosinotto et al. 2021, 2022). Cytochrome Oxidase subunit 1 (COI1) was chosen as the mitochondrial gene, and species-specific primers were designed based on previously published COI1 sequence of *Strix aluco* (GenBank: KF452084.1). Specificity was verified by the presence of a single narrow peak in melting curve analysis, as well as by the presence of a single product of the expected size (79 bp) on agarose gel. COI1 and sfsr/3 reactions were performed on the same plate and each sample was measured in triplicate. Morph and year were always balanced within each plate. To normalize the amplification ratio between different plates, we used a DNA sample being a pool of 4 nestlings as a reference sample (ratio = 1) on every plate. One standard curve (2.5–40 ng) was included on each plate and used to assess qPCR efficiency. One inter-plate standard sample was also run on every plate (CV = 10.4%). qPCR assays were conducted in a total volume of 12 μl containing 5 μl of DNA sample (10ng of DNA) and 7 μl of reaction mix containing primers (forward and reverse) at a final concentration of 300 nM for sfsr/3 and 100 nM for COI1, and Sensifast SYBR®Low-ROX Mix (Bioline). qPCR assays were performed on a 384-QuantStudioTM 12K Flex Real-Time PCR System (Thermo Fisher) and qPCR conditions were 3 min at 95°C, followed by 40 cycles of 5s at 95°C, 25s at 60°C. The melting curve program was 15s at 95°C, 1min at 60°C, 0.1°C/s increase to 95°C, and then hold 15s at 95°C. qPCR efficiencies of control and mitochondrial genes were 97.1 ± 3.1% and 98.3 ± 1.8%, respectively. The mtDNAcn was calculated as (1+EfCOI1) ^ΔCqCOI1^ / (1+Efsfsr/3) ^ΔCqsfsr/3^, Ef being the amplification efficiency and ΔCq being the difference between the Cq values of the reference sample and the sample of interest. Repeatability of mtDNAcn measurements based on the triplicates was high *R* = 0.976 (CI_95%_ = [0.967; 0.982], *n* = 405).

#### Oxidative stress biomarker: reactive-oxygen metabolites (ROMs)

In years 2017–2019, one blood sample per individual was also collected using heparinized capillaries and stored in cold for maximum 12 hours before to be centrifuged at 2375 g for 5 minutes to separate plasma from blood cells (Karell et al. 2011b). The plasma was then stored at -80°C. The concentration of ROMs in the plasma was measured using the d-ROM test (5 µL of plasma, Diacron International, Italy) in duplicates. On every plate the same pooled sample was used to calculate inter-assay variation as long as blank and calibrator, following the manufacturer instructions. The d-ROMs test measures mostly hydroperoxides (ROOH) as a marker of potential oxidative stress and has been validated and extensively used in the past decade in birds (Costantini 2016). ROMs concentration is expressed as mg H_2_O_2_/dl plasma and inter-assay (inter-plate) variation was 5.9% while intra-individual variation based on duplicates was 8.6 ± 2.7%.

#### Thyroid hormones

The plasma obtained from blood samples collected in 2019, stored and separated as described above for ROMs analyses, was also used for measuring thyroid hormones in nestlings (the analysis could only be performed for nestlings from 2019 because of limitation in plasma quantity in other years). The biologically active form triiodothyronine (T3) and its precursor thyroxine (T4) were measured with nano-LC–MS/MS following the protocols in Ruuskanen et al. (2018, 2019). The concentration of T3 and T4 are expressed as pmol/µl.

### Statistical methods

Mitochondrial density and ROMs were analysed on the same adults (on blood and plasma samples respectively) except for one sample that did not work/did not have enough plasma for ROMs analyses and was thus discarded. From both these datasets 1 sample was discarded because it was of 1 female that bred twice in the population, in 2017 and in 2019; the sample from 2019 was therefore excluded from the analyses for both mitochondrial density and ROMs to have only 1 measure per individual. Overall we aimed to sample all the adults in the population but this was not always possible due to methodological constraints in the field. Among the 33 adults considered, for 11 adults it was not possible to sample their partner and thus they are the only representative adult for that territory, whereas the remaining 22 individuals are pairs. Final sample size for mitochondrial density (mtDNA copy number) was thus 33 adults (15males, 18 females; 20 grey, 13 brown) in years 2017–2019 and for ROMs analyses was 32 adults (14 males, 18 females; 20 grey, 12 brown) in years 2017-2019.

Mitochondrial density in nestlings was measured in 101 individuals from 33 families (59 males, 42 females; 52 grey, 49 brown) in years 2016–2019. For ROMs analyses however plasma samples for 2016 were not available and thus only 74 individuals could be tested; of these, two samples did not have enough plasma for analyses and where thus discarded. Thus, the final sample size for ROMs analyses is 72 nestlings from 23 families (44 males, 28 females; 39 grey, 33 brown) in years 2017–2019. Thyroid hormones (T4 and T3) were measured in 2019 only, on 30 nestlings from 16 families. Among these, 16 were grey nestlings (7 females and 9 males) and 14 brown nestlings (8 females and 6 males).

All the analyses were run in R 3.6.1 (R core Team 2019) and RStudio (2019) and graphs are based on least square means, used also to measure standardized effect size *ES* (package “emmeans”; Lenth 2022). We ran linear models for adults (LM) and linear mixed models (LMM) for offspring for all physiological parameters: mitochondrial density, ROMs and thyroid hormones analyses (functions “lmer”, “lme4” package; Bateset al. 2015). All response variables appeared normally distributed, except for mitochondrial density, which was log-transformed to reach normality of the residuals in both adults and offspring models. In all three offspring models “broodID” (i.e. a unique code identifying each nest) was included as random intercept to take into account non-independence of offspring within the same brood.

The fixed factors included in all models were: colour morph (grey or brown), brood size (continuous variable: 0–5 nestlings per nest), and sex (male or female). In the models for mitochondrial density and ROMs sampling year was also included as covariate, to control for different environmental conditions in different years (e.g. temperature and food availability); this was not necessary in thyroid hormone analyses since only 1 year was considered. In all offspring models wing length was included as covariate to control for slight age differences between offspring (i.e. wing length is considered throughout as proxy of age).

Moreover, in the model for adults for ROMs and the two models for offspring mitochondrial density and ROMs, plateID was included as covariate to control for potential technical variation between assays in laboratory analyses (this was not necessary in model for adult mitochondrial density and nestling thyroid hormones as all samples were measured together in the same plate/run. In the model for thyroid hormones in nestlings (both T3 and T4) the time of the day when blood sampling occurred was included as covariate to control for potential circadian variation in hormonal levels. Finally, in both models for mitochondrial density the effect of storage method (ethanol or SET buffer) was also checked by running it as covariate in all the models but it did not affect the results, as previously observed also in analyses for telomere length (see Morosinotto et al. 2021, 2022), and was thus removed from the models.

## Results

### Nestlings

**Fig. 1:**
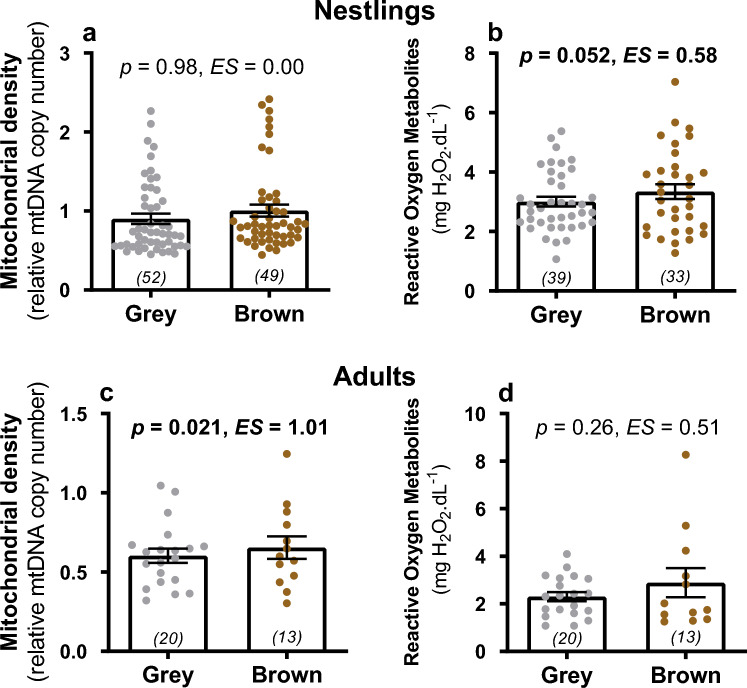
Estimates (least square means) ± SE for effect of morph (grey vs. brown) on mitochondrial density (log transformed) in nestlings (**A**) and adults (**C**) and on reactive-oxygen metabolites (mg H_2_O_2_/dl) on nestlings (**B**) and adults (**D**). Grey and brown dots respectively show raw data for each morph and sample size is presented in parenthesis at the bottom of each column; p refers to the p value presented in Table 1 & 3 and ES refers to the standardized effect size (calculated with emmeans package in R)

Nestling tawny owls did not significantly differ in their mitochondrial density according to colour morph (Fig 1A) or brood size (Table 1A). However, older offspring had lower mitochondrial density than younger ones and variation between years was also evident (Table 1A). There was a non-significant tendency for males to have lower mitochondrial density than females (Table 1A).

**Table 1:** Results of statistical models investigating variation in mitochondrial density (A) and oxidative damage (as mg H_2_O_2_/dl; B) in nestlings

Nestlings									
A) Mitochondrial density (mtDNA copy number; LMM; *n* = 101 in 33 broods)					B) Oxidative damage (ROMs; LMM; *n*=72 in 23 broods)				
Variables	Estimate ± SE	df	*t*	*P*	Variables	Estimate ± SE	df	*t*	*P*
Intercept	0.24 ± 0.49	66.79	0.49	0.625	**Intercept**	**3.08 ± 1.36**	**62**	**2.27**	**0.027**
Morph(brown)	− 0.002 ± 0.08	86.69	− 0.02	0.985	**Morph(brown)**	**0.67±0.34**	**62**	**1.98**	**0.052**
Brood size	0.01 ± 0.08	31.45	0.14	0.893	**Brood size**	**0.47±0.23**	**62**	**2.07**	**0.042**
Sex(male)	− 0.13 ± 0.07	78.82	− 1.72	0.089	Sex(male)	− 0.11 ±0.29	62	− 0.39	0.70
Year(2017)	− 0.09 ± 0.20	29.53	− 0.48	0.637	Year (2018)	− 0.32±0.52	62	− 0.62	0.538
**Year (2018)**	**0.51 ± 0.23**	**34.87**	**2.27**	**0.029**	Year(2019)	− 0.64±0.44	62	− 1.45	0.152
**Year(2019)**	**0.41 ± 0.15**	**28.68**	**2.70**	**0.011**	Plate(2)	− 0.42±0.36	62	− 1.19	0.240
Plate(3)	0.13 ± 0.15	29.13	0.88	0.387	Plate(3)	− 0.64±0.46	62	− 1.41	0.165
Plate(4)	0.22 ± 0.16	30.76	1.44	0.159	Plate(4)	− 0.88±0.62	62	− 1.42	0.161
**Wing**	− **0.005 ± 0.002**	**87.87**	− **2.36**	**0.020**	Wing	− 0.007±0.007	62	− 1.02	0.312
Random factors (variance)					Random factors (Variance)				
BroodID 0.075; Residuals 0.094					BroodID 0.00; Residuals 1.361				

Mitochondrial density was log-transformed. Grey, female, years 2016 (for model A) and 2017 (for model B) and plate 2 (model A) and plate 1 (model B) are used as reference levels for the variables morph, sex, year and plate respectively. Significant effects (*p*-value 0.05 or lower) are highlighted in bold

Brown nestlings showed marginally higher levels of oxidative damage compared to grey ones (Table 1B; Fig. 1B). Nestlings also showed higher oxidative damage when living in larger broods, whereas no statistical differences were observed according to sex, wing length (as proxy for age) or year (Table 1B).

Female tawny owl nestlings had significantly higher levels of both T3 and T4 than male nestlings (Table 2; least square means ± SE T3 female nestlings 4.00 ± 0.485, males 2.65 ± 0.495; T4 females 8.82 ± 0.734, males 6.70 ± 0.754), whereas there were no significant differences according to colour morph (Table 2; least square means ± SE T3 grey nestlings 3.51 ± 0.488, brown 3.14 ± 0.519; T4 grey 7.75 ± 0.733, brown 7.77 ± 0.794). T3 and T4 levels also did not differ according to brood size, wing length, nor hour of sampling (Table 2).

**Table 2:** Results of statistical models investigating variation in thyroid hormones (as pmol/µl) T3 (A) and T4 (B) in nestlings

Nestlings									
A) T3 (LMM; *n*=30 in 16 broods)					B) T4 (LMM; *n*=30 in 16 broods)				
Variables	Estimate ± SE	df	*t*	*P*	Variables	Estimate ± SE	df	*t*	*P*
Intercept	2.61 ± 3.48	17.67	0.75	0.462	**Intercept**	**15.84 ± 5.16**	**14.36**	**3.07**	**0.008**
Morph(brown)	− 0.37 ± 0.53	16.77	− 0.71	0.489	Morph(brown)	0.03 ± 0.93	22.44	0.03	0.978
Brood size	− 0.72 ± 0.60	19.42	− 1.20	0.245	Brood size	− 0.73 ± 0.91	17.73	− 0.81	0.431
**Sex(male)**	− **1.34 ± 0.49**	**13.99**	− **2.73**	**0.016**	**Sex(male)**	− **2.11 ± 0.91**	**16.17**	− **2.33**	**0.033**
Wing	0.006 ± 0.02	24.0	0.32	0.752	Wing	− 0.03 ± 0.03	19.83	− 1.01	0.323
Hour sampling	0.25 ± 0.15	9.87	1.64	0.133	Hour sampling	0.03 ± 0.21	8.86	0.17	0.872
Random factors (variance)					Random factors (Variance)				
BroodID 2.196; Residuals 1.024					BroodID 2.987; Residuals 3.994				

Grey and female are used as reference levels for the variables morph and sex respectively. Significant effects (*p*-value 0.05 or lower) are highlighted in bold

### Adults

Adult brown tawny owls had significantly higher mitochondrial density than grey owls (Table 3A, Fig. 1C). Higher mitochondrial density was also observed when adults raised larger broods, and mitochondrial density varied among years (with higher and lower levels in 2018 and 2019 respectively compared to 2017; Table 3A), while no clear difference was observed according to adult sex (Table 3A). Oxidative stress, measured as ROMs, was not significantly explained by colour morph (Fig. 1D), brood size, nor by the other covariates (Table 3B).

**Table 3:** Results of statistical models investigating variation in mitochondrial density (A) and oxidative damage (B) in adults

Adults							
A) Mitochondrial density (mtDNA copy number; LM; *N*=33)				B) Oxidative damage (ROMs,;LM; *N*=32)			
Variables	Estimate ± SE	*t*	*P*	Variables	Estimate ± SE	*t*	*P*
Intercept	− **1.03 ± 0.25**	− **4.18**	**0.0003**	Intercept	1.67 ± 1.32	1.27	0.217
**Morph (brown)**	**0.29 ±0.12**	**2.45**	**0.021**	Morph(brown)	0.78 ± 0.67	1.16	0.258
**Brood size**	**0.15 ± 0.07**	**2.34**	**0.027**	Brood size	0.43 ± 0.36	1.20	0.243
Sex(male)	0.004 ± 0.10	0.04	0.968	Sex(male)	− 0.22 ± 0.59	− 0.38	0.710
**Year (2018)**	**0.34 ± 0.15**	**2.33**	**0.028**	Year (2018)	0.018 ± 0.85	0.02	0.984
**Year (2019)**	− **0.43 ± 0.14**	− **3.07**	**0.005**	Year(2019)	− 0.37 ± 0.74	− 0.51	0.618
				Plate(2)	− 0.70 ± 0.62	− 1.13	0.271
				Plate(4)	− 0.64 ± 0.81	− 0.78	0.442

Mitochondrial density was log-transformed. Grey, female and 2017 are used as reference levels for the variables morph, year and sex respectively. Plate 1 is used as reference level for plate in model B). Significant effects (*p*-value 0.05 or lower) are highlighted in bold

## Discussion

Our results suggest that there is large variation in mitochondrial density among individuals across life stages, which points out that such variation may be directly linked with growth and work load and hence directly associated with life history trade offs and resource allocation strategies. Indeed we find that colour morphs, which have been found to adopt different life history strategies both during growth (Piault et al. 2009, Morosinotto et al. 2020) and as adult (Emaresi et al. 2014, Bucciolini et al. 2025), differ in mitochondrial density and dROM in the blood. In particular, our data show that in tawny owls mitochondrial density (i.e. the number of mitochondria per cell) is elevated in breeding brown adults compared to grey ones, but that there are no significant differences in oxidative stress between morphs. On the other hand, in growing offspring there are no differences in mitochondrial density (i.e. number of mitochondria per cell) nor thyroid hormones, but a tendency for higher levels of oxidative stress in brown compared to grey individuals. Hence, these physiological markers tend to vary substantially between individuals and to be morph-specific as well as life stage specific.

We found that mitochondrial density was significantly higher in brown adults compared to grey during breeding. Although estimating mitochondrial density does not allow to predict mitochondrial aerobic metabolism and efficiency, it has been shown to correlate with aerobic metabolism (Stier et al. 2019; Cossin-Sevrin et al. 2022) and could be linked to differential reproductive effort among the morphs (i.e. brown individuals investing more than grey ones; Emaresi et al. 2014; Morosinotto et al. 2020). Indeed, individuals with higher reproductive effort often need more energy and a higher number of mitochondria could allow the increase of ATP production. The positive association between brood size and mitochondrial density observed here support this hypothesis and previous studies have shown a link between reproductive investment and mitochondrial density (Garratt al. 2013; Zhang et al 2018). For example, lactating mice (*Mus musculus*) have higher mitochondrial density than non-reproducing females (Garratt et al. 2013) and mice in good body condition (i.e. trained in running during gestation) had higher reproductive investment and mitochondrial density (Zhang et al. 2018). The higher mitochondrial density we observed in brown individuals could potentially increase ROS production and oxidative stress (Passos et al. 2007), but we observed no significant difference between morphs in ROMs levels. Yet, it is worth noting that the effect size for the morph-effect on ROMs in adults was in the same direction and magnitude that the one observed in nestlings, and thus a larger sample size in adults might be required to reach statistical significance. Unfortunately antioxidant defenses could not be measured in the present study due to methodological constraints but it would be an important addition to have a more complete overview of the physiological profile of tawny owls. Oxidative stress dynamics in this study system are still understudied but the previous studies suggested that brown adults have overall lower total glutathione (a key intracellular antioxidant molecule) levels than grey adults (Emaresi et al. 2016). Lower glutathione levels could suggest that brown adults are less capable of mounting a strong antioxidant defense when exposed to oxidative stress and may thus incur in stronger damage. An increase in metabolic rate or mitochondrial density (to support higher reproductive investment) does also not necessarily increase ROS production (Salin et al. 2015) and/or an up-regulation of antioxidant defenses during reproduction could occur (Garratt et al. 2013; Blount et al. 2016).

The relationship between colour-morph and physiology in nestlings markedly differ from what was observed in adults. Indeed, we observed no significant difference in mitochondrial density between brown and grey nestlings, which also fits with the observation that thyroid hormones did not significantly differ between the two morphs. Yet, brown nestlings tended to have significantly higher levels of oxidative stress than grey ones. Higher oxidative stress in brown nestlings may be linked to their faster growth since they are usually heavier at fledging (Morosinotto et al. 2020) and grow bigger when fed ad libitum (Piault et al. 2009). Fast growth has been suggested to carry an oxidative cost (Smith et al. 2016) potentially through its link with higher mitochondrial efficiency (Salin et al. 2019). Accordingly, a recent experiment on damselfly (*Lestes viridis*) larvae showed that fast compensatory growth is inducing an increase in oxidative damage that could be prevented by a reduction in mitochondrial efficiency (Janssens and Stoks 2020). Consequently, it would be very relevant to explore if brown tawny owl nestlings could have more efficient mitochondria (since they do not appear to have higher density) enabling them to grow faster than grey ones, but at the expense of higher oxidative stress.

Our results thus suggest that genetically-determined distinctive phenotypes exhibit different physiological profiles. The question remains open whether these physiological traits could be linked with the trade-offs existing between the energy necessary for growth and reproduction vs. survival. Mitochondrial density, as well as oxidative stress dynamics, varied according to both the life stage considered and the colour morph and the patterns here observed could be linked to morph-specific life-history traits. Brown breeding adults have higher mitochondrial density and also seem to invest more in reproduction (Emaresi et al. 2014; Karell et al. 2017; Morosinotto et al. 2020), while brown nestlings tend to have higher oxidative stress and are known to grow bigger at fledging, which could be mediated by offspring ontogeny and/or differential parental care provided by brown parents (Piault et al. 2009; Emaresi et al. 2014; Morosinotto et al. 2020). Our results, albeit correlative in nature, suggest that these physiological traits could be associated with life–history strategies adopted by the morphs. Further studies should experimentally manipulate reproductive effort and growth conditions, to better understand the link between physiological profiles of different phenotypes and the life–history strategies adopted.

## Supplementary Information

Below is the link to the electronic supplementary material.

Supplementary file1 (XLSX 29 KB)

Supplementary file2 (XLSX 31 KB)

## Data Availability

All the data will be uploaded on a public repository upon acceptance.

## Code availability

The code used in the analyses will be uploaded on a public repository upon acceptance.
